# Opportunities for Maximizing the Dietary Quality of Fad Diets

**DOI:** 10.3390/nu15214526

**Published:** 2023-10-25

**Authors:** Jessica M. Phelan, Jillian M. Joyce, Katherine Bode, Sara K. Rosenkranz

**Affiliations:** 1 Department of Food Nutrition Dietetics and Health, Kansas State University, Manhattan, KS 66506, USA; 2 Department of Health and Human Performance, Fort Hays State University, Hays, KS 67550, USA; 3 Department of Nutritional Sciences, Oklahoma State University, Stillwater, OK 74078, USA; jill.joyce@okstate.edu (J.M.J.);; 4 Department of Kinesiology and Nutrition Sciences, University of Nevada, Las Vegas, NV 89154, USA; sara.rosenkranz@unlv.edu

**Keywords:** dietary quality, college student, fad diet, healthy eating index, dietary analysis

## Abstract

The quality of American diets, measured by the Healthy Eating Index (HEI), has remained stable and low since 2005. The Dietary Guidelines for Americans 2020–2025 call for research analyzing dietary patterns to determine how guidelines might be altered to increase healthy eating. The present paper seeks to determine the dietary quality of popular fad dietary patterns among Americans. A definition of “fad diet” was created, and Google Trends© was searched for popular diets to determine popular dietary patterns based on the fad diet definition. Finally, eight dietary patterns were identified for inclusion. One-week sample menus were created for each dietary pattern, maximizing alignment with the DGAs but staying within the dietary pattern parameters, and then scored according to the HEI 2015 to determine the dietary quality. Total HEI scores ranged from 26.7 (Carnivore) to 89.1 (Low-FODMAP); the six highest total HEI scores were in the range of 77.1–89.1 out of 100 points. This analytical approach showed that some of the included popular fad dietary patterns have the potential to attain a high dietary quality. Rather than suggesting one “best” diet or dietary pattern, there is opportunity to maximize dietary quality in the context of dietary patterns that are considered fad diets.

## 1. Introduction

The 2020–2025 version of the Dietary Guidelines for Americans (DGA) [1] revealed that, despite continual public health nutrition education, policy, and programmatic efforts, Americans are not adhering to the dietary guidelines [2]. Measured yearly in the period of 2005–2016, scores were in the range of 56–60 out of 100 on the Healthy Eating Index (HEI), which measures dietary quality or healthfulness of dietary patterns based on adherence to the DGAs [3]. These scores suggest that a relatively poor adherence has persisted over a long period of time [4]. Additionally, around 17% of adults in 2015–2018 reported following some sort of special diet [5]. To determine why Americans have maintained low HEI scores over the past several DGA releases, the Dietary Guidelines Advisory Committee (DGAC) suggests that researchers take into consideration existing dietary patterns that influence dietary consumption to better manage and treat diet-related diseases. For the purpose of this study, we focus on the less scientifically accepted dietary patterns that Americans might be consuming, sometimes referred to as “fad diets”, rather than the dietary patterns that are scientifically well-established, and generally regarded as health-promoting, such as the Mediterranean diet or Dietary Approaches to Stop Hypertension diet. Little research has been conducted to determine the potential for high dietary quality among fad dietary patterns.

Research suggests that the Westernized diet, followed by many Americans, is characterized as high in calories, saturated and trans fats, sugar and sodium, with large portions, and low in fruits, vegetables, and fibers [6]. Furthermore, around 80% of Americans habitually under consume fruits, vegetables, and dairy/dairy alternatives as compared to the DGA recommendations [1]. Americans also overconsume refined grains and animal proteins, added sugars, saturated fats, sodium, and alcoholic beverages [1]. Added sugars and saturated fats account for around 13% and 11% or more of the total calories allocated per day, respectively, for the average American, well above the 15% combined total calorie allotment for these types of foods [1].

Overall, despite the continual refinement of the DGAs to reflect current nutritional needs, adherence to the guidelines remains problematic, and the quality of the American diet is persistently within the “needs improvement” HEI scoring range of 51–80 [7]. Given the importance of dietary quality for long-term health outcomes, understanding the dietary quality of popular dietary patterns is critical; this understanding may offer insights into what Americans are actually eating, thereby allowing the assessment of areas of poor adherence to dietary guidelines. Therefore, the primary purpose of the current study is to determine the most “popular” fad dietary patterns in the United States. The secondary purpose is to evaluate the dietary quality of relevant popular fad dietary patterns as compared to the DGAs using the HEI. Finally, we synthesize the results across the included fad dietary patterns in order to offer insights regarding opportunities for maximizing the dietary quality.

## 2. Methods

### 2.1. Determination of a Working Definition of a Fad Diet

For the purposes of this study, we defined the term “dietary patterns” according to the DGA Committee 2020 definition, “the quantities, proportions, variety, or combination of different foods, drink, and nutrients in diets, and the frequency with which they are habitually consumed” [2]. To determine a comprehensive working definition of a “fad diet”, two independent researchers compiled a database of definitions and diet attributes from peer-reviewed sources (29%), textbooks (21%), and websites, reports, popular books, and blogs (50%), as shown in Table 1. Common themes appearing across numerous database entries derived from using the search term, “fad diet”, were identified as indicated in Table 1. The common themes that appeared the most frequently in database entries included (1) lack of physical activity, or no component related to encouraging physical activity; (2) rigidity, where intake of certain foods was required; (3) marketing and endorsements by celebrities; (4) restriction, where the complete omission of foods or food groups was required; and (5) rapid timeframes for weight loss. The frequency of these themes was as follows: lack of physical activity (36%), rigidity (50%), marketing (57%), restriction (64%), and time (92%). Using these themes as a framework, the following working definition of a fad diet was established for the purpose of the current study: a pattern of eating aimed at rapid, unsustainable weight loss that may severely restrict calories and nutrients, by omitting foods or entire food groups, and/or recommending rigid eating patterns, restricting physical activity, or indicating the lack of necessity for physical activity, where reported results are not evidence-based nor intended for the treatment of medical conditions and are marketed through endorsements from authority figures or celebrities.

### 2.2. Selection of Fad Diets for Analysis

**Figure 1:**
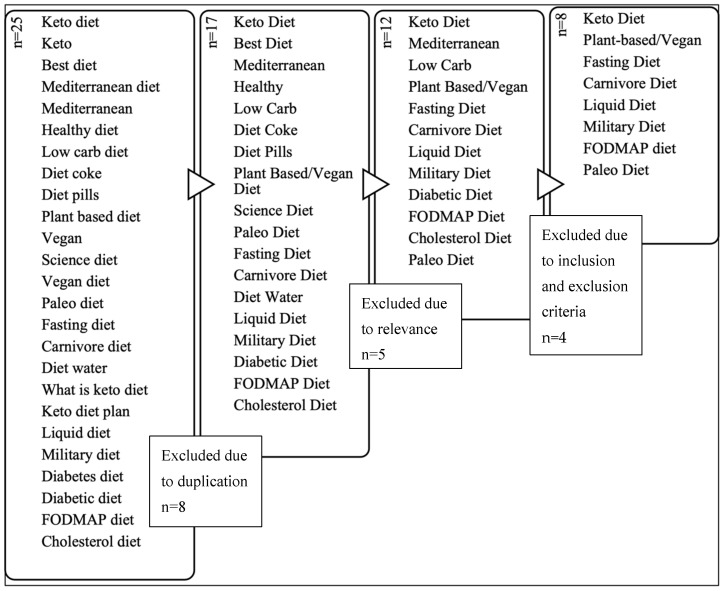
Process of selecting the included dietary patterns.

Google Trends^©^ was then used to delimit the “popular” dietary patterns for inclusion in the current study. For the purpose of this paper, “popular” was defined as the most widely searched terms or phrases related to fad diets. First, the term “diet” was searched, and results were filtered to include only those terms or phrases that were searched between 1 January and 7 October 2020 at 12:30 pm. The results showed the top 25 searched terms or phrases (*n* = 25) that included the keyword “diet”. To ensure the continued relevance of these fad diets, an identical search was conducted on 31 January 2022. The results were filtered to include the terms or phrases that were searched between 7 October and 31 January. The results were reviewed to identify any new dietary patterns that were not previously included. Terms or phrases were eliminated due to duplication (*n* = 8) and/or relevance (*n* = 5) where diets were not for human consumption, were not a dietary pattern, or included supplements only. The inclusion and exclusion criteria were derived from the agreed upon working definition of “fad diets”. The inclusion criteria were dietary patterns that (1) suggested rapid, unsustainable weight loss; (2) severely restricted daily caloric intake; (3) omitted entire food groups or recommended rigid eating patterns outside of the Acceptable Macronutrient Distribution Ranges (AMDRs) for carbohydrates, fats, and protein, 45–65%, 20–35%, and 10–35% of the total energy intake, respectively [21]; (4) restricted or diminished the importance of physical activity; and/or (5) suggested results that were not supported by peer-reviewed research evidence. The exclusion criteria included any dietary patterns that (1) had accepted credible peer-reviewed evidence available to show a sustainable weight loss and/or health benefits; (2) allowed flexibility within the eating pattern; (3) did not severely restrict calories and/or nutrients; and (4) suggested physical activity to accompany the dietary pattern. After applying the selection criteria to the remaining 12 terms or phrases, the following dietary patterns (*n* = 8) were included for evaluation and synthesis: Ketogenic Diet, Plant-based/Vegan Diet, Fasting Diet, Carnivore Diet, Liquid Diet, Military Diet, Low-FODMAP Diet, and the Paleo Diet. The process for selecting the final eight dietary patterns is shown in Figure 1. These eight dietary patterns are referred to as popular fad dietary patterns hereafter.

### 2.3. Operationalization of Popular Fad Dietary Patterns

In order to operationalize each popular fad dietary pattern for subsequent evaluation and synthesis, an agreed upon/mutual understanding of each popular fad dietary pattern was determined. Two independent researchers identified the parameters of the popular fad dietary patterns from peer-reviewed sources, and when no peer-reviewed sources were available, information was obtained from websites, reports, popular books, or blogs to determine the specifics of each popular fad dietary pattern, as well as the potential mechanisms of action related to weight loss and/or health outcomes. Non-peer-reviewed data were used only when peer-reviewed sources were not available. The priority for the use of evidence was as follows: peer-reviewed sources; governmental websites and reports; and websites, popular books, and blogs. The popular fad dietary pattern definitions (Table 2) included specific parameters, such as calorie limits, micronutrient compositions, dietary components that were restricted, required supplements, and required special food products. Any disagreements regarding these parameters were first discussed by two researchers (J.P. and K.B.) and additional researchers (S.K.R. and J.J.) were included in discussions when needed in order to arrive at one standardized definition for each popular fad dietary patterns.

### 2.4. Creation of Menus for Analysis

One-week menus utilizing the operationalized parameters of the popular fad dietary patterns were created using a process that is similar to a clinical dietitian’s process (created by J.J. and K.B.) for creating a meal plan for a patient by using the specified food group servings spread out into meals and snacks throughout the day to create a daily meal pattern, followed by menu-planning principles to specify food items that fulfill the food group servings in the meal pattern (i.e., start with planning the entrée of the main meals then move on to planning sides and finally snacks; consider varied aesthetic elements, like colors and textures across meals and days; and ensure foods within meals and snacks complement one another).

**Figure 2:**
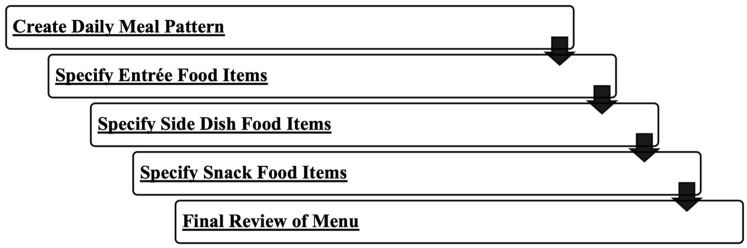
Process of creating menus for analysis.

As seen in Figure 2, first, the recommended servings of each food group from Table 3 were distributed across three meals and up to three snacks daily to create a daily meal pattern. Second, the entrées (protein and grain) for lunch and dinner, followed by breakfast, were planned to ensure variety (color, texture, temperature, flavor, and visual appeal) from day to day and meal to meal while meeting the food group serving recommendations. Then, the vegetable and fruit side dishes for the main meals were planned to ensure variety across days and meals, meeting food group serving recommendations and esthetically complementing the entrée. Next, the snacks were planned to ensure variety across days and meals, meeting food group needs, and for snack items to esthetically complement one another. Once the meal plan draft was created, the researchers reviewed them and ensured that all food group and subgroup (i.e., protein types, vegetable types, and carbohydrate types) requirements were met for the specific diet.

To ensure that menus were created to comply with the DGAs to the extent possible, maximizing dietary quality within the rules of each popular fad dietary pattern, a DGA-compliant meal pattern and menu were first created and were then used as the template for subsequent popular fad dietary pattern menus, modified according to the differences in the food group requirements shown in Table 3. This process helped to reduce potential researcher bias regarding the popular fad dietary patterns that could result in lower dietary quality scores for the popular fad dietary pattern menus.

### 2.5. Data Analysis: Part 5

Dietary intake data were analyzed using the Automated Self-Administered 24 h (ASA24^®^) Dietary Assessment Tool, version (2021), developed by the National Cancer Institute, Bethesda, MD [34]. Each day of the sample menus was entered in the ASA24^®^ software, and specific foods, portion sizes, and drinks and condiments were indicated. Once each menu was entered, the data were extracted and analyzed using the statical software SAS, version 8, copyright © 2022 [35]. The data were analyzed according to the NCI SAS code provided for all researchers to determine HEI-2015 scores in the range of 0–100, where 0 indicates a low adherence to the DGAs and 100 indicates full adherence. The following variables were analyzed: total vegetables, greens and beans, total fruit, whole fruit, whole grain, dairy/dairy alternatives, total protein foods, seafood and plant protein, fatty acid ratio, sodium, refined grains, saturated fat, added sugar, and total HEI-2015 score [36]. The outcome variables were analyzed based on their categorization regarding adequacy (total vegetables, greens and beans, total fruit, whole fruit, grains, dairy/dairy alternatives, total protein foods, seafoods and plant protein, and fatty acid ratio), where a higher intake is preferable, or moderation, where a lower intake is preferable (sodium, refined grains, saturated fats, and added sugar).

**Figure 3:**
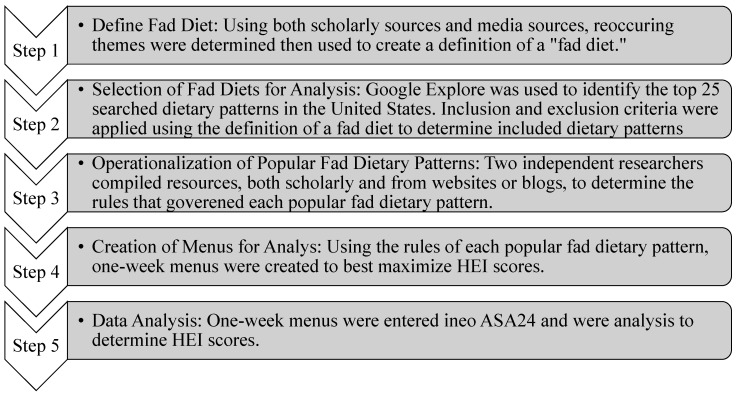
Methods for the selection of dietary patterns for the analysis.

Micronutrient analyses were determined by calculating the means and standard deviations per nutrient and comparing those with the Dietary Reference Intakes (DRIs): Recommended Dietary Allowances (RDAs) [37]. Recommendations for the 19–50 y age group for both males and females were used for comparisons. The micronutrient recommendations were determined to have been met if the means and standard deviations met or exceeded the RDAs for both males and females. Figure 3 shows the methods for selecting and analyzing the included fad dietary patterns.

## 3. Results

### 3.1. Popular Fad Dietary Patterns’ Definitions

The Ketogenic Diet, Plant-based/Vegan Diet, Fasting Diet, Carnivore Diet, Liquid Diet, Military Diet, Low-FODMAP Diet, and the Paleo Diet were identified for inclusion in the current study based on the inclusion and exclusion criteria for the fad diet themes. In order to ensure a shared understanding of each popular fad dietary pattern, the following operational definitions were determined for each popular fad dietary pattern along with the proposed mechanisms of action for weight loss.

### 3.2. Ketogenic Diet

The Ketogenic Diet was popularized in the 1920s by Russell Wilder as a treatment for epilepsy, where the restriction of carbohydrates, used as a medical nutrition therapy, was used to alter energy metabolism and neurotransmitter function [38]. This diet is characterized by an extremely low carbohydrate consumption (<50 g/day), moderate protein consumption (~20% of total caloric intake/day), and high fat intake (~70% of dietary intake/day) [22]. Overall, the goal of the Ketogenic Diet is to follow any pattern of food consumption that would result in nutritional ketosis [38]. Due to the omission of entire food groups and recommendations of rigid eating patterns outside the AMDRs, the Ketogenic Diet is considered a popular fad dietary pattern for the purposes of the present study. The Ketogenic Diet has gained popularity due to its metabolic effects and has been repurposed from its original intended use as a medical nutritional therapy for use as a method for weight loss. This restrictive dietary pattern may not be easily maintained in the long term; therefore, once dietary intake returns back to “normal” and carbohydrates are increased with more typical fat and protein amounts, weight may be regained [39].

The mechanism of action for this dietary pattern is centered around ketosis. When carbohydrates are absent from or lacking in the diet, other substrates are used to supply the body with energy. Glucose stores become insufficient for energy needs, thereby influencing energy availability for the central nervous system (CNS) [40]. Insulin production decreases, while glucagon production increases, signaling the liver to decrease lipogenesis and increase the synthesis of mitochondrial fatty acids [40]. Ketone bodies, namely acetoacetate and beta-hydroxybutyrate, are formed by the incomplete breakdown of fatty acids due to the insufficient amounts of oxaloacetate. As ketone bodies are increased and become the predominant fuel source, nutritional ketosis is achieved. Some published research outcomes associated with a Ketogenic Diet include weight loss, increased insulin sensitivity, potential cancer prevention, and the treatment of neurological disorders, as well as negative side effects, including muscle cramps, lethargy, micronutrient deficiencies, hepatic insulin resistance, increase in oxidative stress, decreased production of certain gut microbiota, vomiting, abdominal pain, and constipation [22]. This pattern has been shown to reduce high blood pressure, decrease blood plasma triglycerides, lower glycosylated hemoglobin (HbA1c), reduce postprandial glycemic response, produce an anti-inflammatory effect, and enhance fat oxidation [22,39].

### 3.3. Plant-Based/Vegan Diet

Plant-based eating is a broad category of eating patterns centered around consuming mostly plant-based foods [41]. Generally, plant-based eating comprises diets that are void of animal flesh foods and where the majority of nutrients and calories are obtained from plants [23]. While plant-based diets are generally perceived as healthful, the rigidity and restriction and/or omission of foods within the protein food group are present; therefore, they fit our inclusion and exclusion criteria for a popular fad dietary pattern. Plant-based diets are generally rich in beans, legumes, nuts, soy, fruits, vegetables, and whole grains, as well as fibers, phytochemicals, and healthy fats [23].

The increased consumption of predominately unprocessed plant-based foods may be associated with weight control due to overall calorie reduction, increased satiety through increased fiber intake, and a greater overall volume of lower calorie/higher water volume content foods. Many other beneficial aspects of a plant-based diet are thought to be due to increased phytochemical, antioxidant, fiber, and micronutrient consumption, as well high nutrient density. Some published research outcomes associated with plant-based/vegan diets include improved HbA1c levels, greater weight loss, and decreased serum low-density lipoproteins (LDL) levels and total cholesterol [21].

### 3.4. Fasting Diet

The Fasting Diet, also known as intermittent fasting, is an umbrella term used to describe heterogeneous dietary patterns where food is completely restricted for specified periods of time followed by periods of normal/typical eating [25]. This process is repeated over time until the desired health effects are reached. Alternate day fasting consists of one day of fasting followed by one day of eating normally or feasting [25,42]. Modified fasting, also known as intermittent energy restriction, is based upon the alternate day fasting method of two days of fasting per week followed by five days of normal eating. However, rather than completely abstaining from any caloric consumption, overall intake is limited to ~20–25% of the total energy needs on fasting days [25]. Periodic fasting is characterized by numerous days of consecutive fasting per month ranging from two days to many [42]. In some cases, fasting may not mean consuming a balanced and healthful diet; rather, it compresses or alters the eating time window.

In other situations, fasting results in omitting entire food groups and extreme caloric reduction. Weight loss resulting from following this dietary pattern may be attributed to reduction in caloric intake and improved insulin sensitivity [43]. In a previous review, Rynderes and colleagues sought to identify the current evidence on intermittent energy restriction as a treatment for overweight and obesity, reviewing studies (*n* = 11) of at least 8-weeks in duration where fasting was compared to a control that included continuous energy restriction. Nine of the eleven included studies suggested that both strategies produced similar weight and fat loss; therefore, the authors concluded that both fasting and continuous calorie restriction are potential methods for the treatment of overweight and obesity.

### 3.5. Carnivore Diet

The Carnivore Diet, which gained popularity in the late 2010s, is a diet that consists of eating only animal/meat products, namely red meats, without any other sources of food, and without intentional restriction of calories [26]. Although this diet suggests strictly eating meat, it is well established that the transition period between other dietary patterns and the Carnivore Diet is difficult; therefore, many diet followers might incorporate other food sources into the diet [26]. There is little to no peer-reviewed evidence over the Carnivore Diet, specifically, and studies where only animal flesh is consumed are absent. The Carnivore Diet suggests omitting entire food groups and has rigid recommendations outside of the AMDRs; therefore, this dietary pattern is considered a popular fad dietary pattern.

The potential mechanistic rationale behind the Carnivore Diet includes evidence that protein intake promotes satiety, thereby encouraging caloric restriction and the maintenance of lean tissue during weight loss [44]. The documented outcomes of high-protein diets, not specific to the Carnivore Diet, include reductions in C-reactive protein, the mitigation of sarcopenia in older adults, increased cholesterol levels, decreased HDL/LDL ratio, and increased probability of colon and breast cancer [45,46,47].

### 3.6. Liquid Diet

The Liquid Diet is a diet consisting only of liquids, allowing no intake of solid foods [28]. Examples of foods permitted on a liquid diet are water, fruit juices without pulp, bone broth, miso, strained vegetable juice, and 100% fruit juice popsicles [48]. Generally, Liquid Diets are intended for the short term, and used pre- and post-surgery, where an empty colon is necessary [28]. The Liquid Diet has been used as a quick weight loss method, which is not the original purpose of the diet. When used in a weight loss context, a Liquid Diet severely restricts caloric intake, omits entire food groups, has rigid recommendations well outside of the AMDRs, and is not supported by research. Some effects of the Liquid Diet are temporary weight loss due to overall reduction in highly calorically dense foods and the absence of food residues in the intestines, low vitamin and mineral intake, potential nutrient deficiencies, and fatigue.

### 3.7. Military Diet

The Military Diet is advertised as a low-calorie diet that includes foods designed to increase weight loss without slowing down metabolic processes [30]. This diet consists of three days of planned meals, as shown in Table 4, followed by four days of normal eating until the desired results are achieved. Alternate meal plans are provided for those following a plant-based diet. The Military Diet is characterized by extreme caloric reduction and rigid eating patterns outside of the AMDRs. The proposed mechanisms of action are extremely low caloric consumption and low carbohydrate consumption. Low carbohydrate consumption leads to lowered glycogen stores, and since water is stored with glycogen, reduction in glycogen may also lead to a lowered body water content [49]. Therefore, weight loss tends to occur due to reduced water weight in the initial stages of the diet. Some of the effects of rapid weight loss diets are reductions in fat mass, quick weight loss due to caloric restriction and loss of stored water, and potential reductions in fat-free mass [50].

### 3.8. Low-FODMAP Diet

The FODMAP Diet, better known as the Low-FODMAP Diet, an acronym for fermentable oligosaccharides, disaccharides, monosaccharides, and polysaccharides, was originally created as a way to control gastrointestinal distress and relieve symptoms related to irritable bowel syndrome (IBS) and small-intestine bacteria overgrowth (SIBO) [32]. The Low-FODMAP Diet suggests the extreme restriction of highly fermentable oligosaccharides, disaccharides, monosaccharides, and polysaccharides, which are found in carbohydrate-rich foods, such as fruits, vegetables, dairy, and grains, until the symptoms of IBS, SIBO, and other gastrointestinal issues subside [31]. Once symptoms have lessened, carbohydrate-rich foods are slowly reintroduced into the diet over time. This type of diet is recommended for those who have symptoms suggestive of gluten or carbohydrate intolerance as a way to identify the foods that provoke problematic symptoms. The Low-FODMAP Diet is sometimes repurposed from its original intent as a quick weight-loss diet, omitting entire food groups. The proposed mechanism of action is associated with decreased short-chain carbohydrate consumption, resulting in a lower fermentation of fibers, subsequently reducing gas production [41]. Additionally, the restriction of FODMAP foods may reduce the total caloric intake, potentially leading to weight loss. Research outcomes associated with the low-FODMAP diet include the treatment of IBS and other IBS-like conditions, reduction in gastrointestinal distress, and short-term weight loss [51].

### 3.9. Paleolithic Diet

The Paleolithic Diet, sometimes referred to as the “Paleo Diet”, “Cave-Man Diet”, or a hunter–gatherer diet, is a way of eating that emphasizes consuming foods that were readily eaten in the Paleolithic era [33]. Specifically, the Paleolithic Diet restricts the consumption of grains, legumes, peanuts, peas, dairy products, refined sugars, artificial sweeteners, salt, potatoes, trans fats, vegetable oil, and highly processed foods. It also emphasizes the intake of fruits, vegetables, lean proteins, seafood, nuts, and seeds [33]. The Paleolithic Diet is characterized as a popular fad dietary pattern due to its omission of entire food groups.

The proposed mechanism of action is through an increased intake of nutrient-dense foods and a decreased intake of nutrient-poor, ultra-processed foods. In a systematic review and meta-analysis, Ghaedi and colleagues reviewed eight eligible randomized controlled studies to determine the influence of a Paleolithic Diet on weight, body composition, circulating concentrations of blood lipids, blood pressure, and inflammatory markers. The health outcomes associated with the Paleolithic Diet in comparison to the control diets included a lowered bodyweight, BMI, and waist circumference. Additionally, Ghaedi and colleagues suggested that improved blood pressure, circulating cholesterol, triglycerides, LDL cholesterol, C-reactive protein, and increased HDL cholesterol could be associated with the Paleolithic Diet, but that results should be interpreted cautiously [52]. Another systematic review and meta-analysis over 31 human intervention trials examined the Paleolithic Diet’s associations to non-communicable diseases, and the results indicated improved bodyweight, BMI, and waist circumference when following the Paleolithic Diet as compared to participants not participating in a diet for weight loss [53].

### 3.10. Healthy Eating Index Scores for Each Dietary Pattern

**Figure 4:**
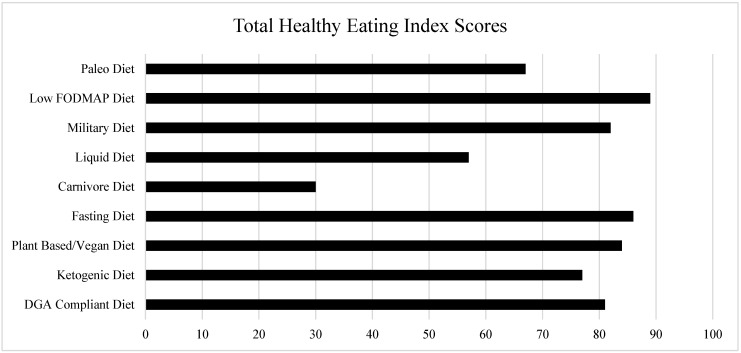
Total Health Eating Index Scores.

The analysis of each popular fad dietary pattern, where adherence to the DGAs was maximized, is shown in Figure 4, revealing total HEI scores ranging from 26.7 (Carnivore) to 89.1 (Low-FODMAP). The six highest total HEI scores were in the range of 77.1–89.1.

**Figure 5:**
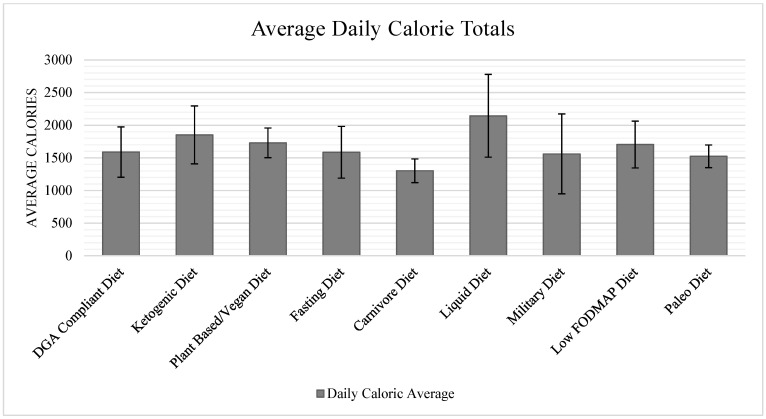
Average daily calorie totals.

Total calorie provision was the highest in the Liquid Diet (2143 kcal/day) and the lowest in the Carnivore Diet (1302 kcal/day). Excluding the Carnivore Diet and Liquid Diet, the remaining seven popular fad dietary patterns’ total calories ranged from 1524 kcal/day for the Paleo Diet to 1852 kcal/day for the Ketogenic Diet. Additionally, Figure 5 shows the average daily calories totals for each of the analyzed dietary patterns. 

Table 5 illustrates the adequacy component scores that were based upon a 5-point scale, where 0 represents non-adherence and 5 represents maximal adherence. Each popular fad dietary pattern—except for the Ketogenic Diet, where carbohydrates were limited, the Carnivore Diet, where plant foods were excluded, and the Liquid Diet—could accommodate the maximal fruit, vegetable, and protein intakes. Overall, adherence was high among the majority of adequacy components for fruits, vegetables, and protein.

Additionally, among the adequacy component scores that were based upon a 10-point scale, where 0 represents non-adherence and 10 represents maximal adherence, overall, whole grains consistently scored low, indicating submaximal adherence among all popular fad dietary patterns. This could be due to how ASA24 classified whole grains versus refined grains according to the theoretical menus. Dairy/dairy alternatives was also an area where scores were submaximal among six of the nine poplar fad dietary patterns. The fatty acids ratios of six of the popular fad dietary patterns scored maximal points, while the DGA Compliant Diet, the Military Diet, and the Liquid Diet did not.

As illustrated in Table 5, for the moderation component scores that were based upon a 10-point scale, where 0 represents non-adherence and 10 represents maximal adherence, sodium scores consistently indicated submaximal adherence among all popular fad dietary patterns, indicating that many of the popular fad dietary patterns provided excessive sodium, even when using the best practices for minimizing sodium content. Refined grain and added sugar scores were high, representing low consumption. Finally, saturated fats scores were generally high among all popular fad dietary patterns, representing a low consumption. Exceptions included the Ketogenic Diet and the Carnivore Diet, due to a high consumption of fats from animal products.

### 3.11. Micronutrient Analysis

**Figure 6:**
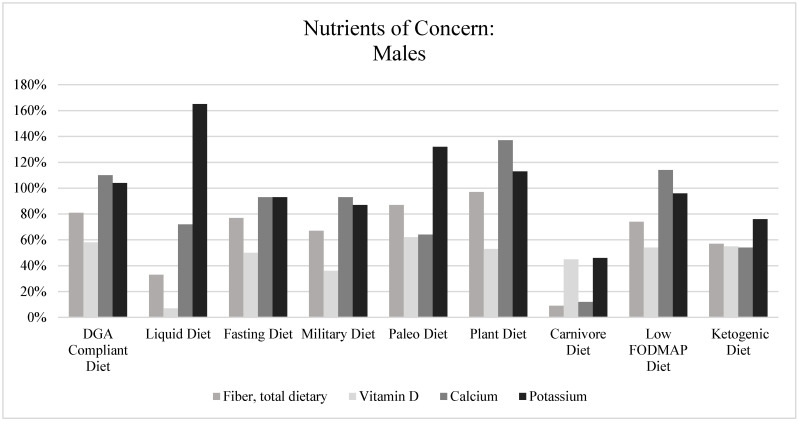
Nutrients of concern: males. Note. Each fad dietary pattern was compared to the RDAs, which were used as the standard, where the RDAs for fiber, vitamin D, calcium, and potassium were 38 g, 15 mcg, 1000 mg, and 3400 mg, respectively. The figure above shows the percentage of attainment of the RDA per nutrient of concern.

**Figure 7:**
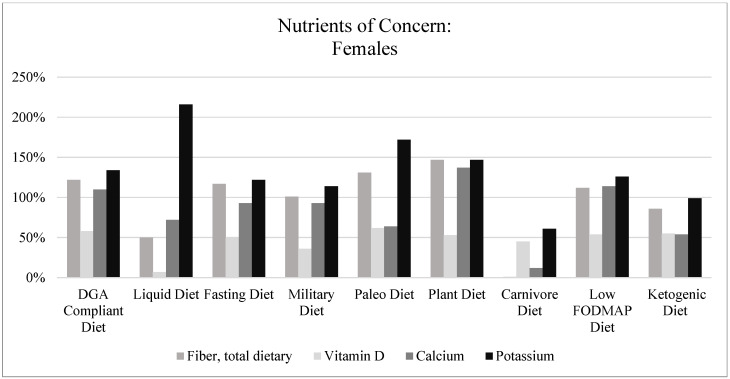
Nutrients of concern: females. Note. Each fad dietary pattern was compared to the RDAs, which were used as the standard, where the RDAs for fiber, vitamin D, calcium, and potassium were 25 g, 15 mcg, 1000 mg, and 2600 mg, respectively. The figure above shows the percentage of attainment of the RDA per nutrient of concern.

Figure 6 and Figure 7 illustrate the micronutrient adequacy of each popular fad dietary pattern as compared to the RDAs for the age range of 19–50 years; specifically, calcium, vitamin D, potassium, and fiber are highlighted due to the DGAs emphasizing these as nutrients of concern for underconsumption for the American population [1]. Calcium was adequate for all fad dietary patterns, except the Paleo Diet, Carnivore Diet, and Ketogenic Diet, and Vitamin D was only adequate for the Paleo Diet. Potassium was adequate in all but the Ketogenic and Carnivore Diets, while fiber was lacking in the Carnivore, Liquid, and Ketogenic Diets. Overall, the Carnivore and Ketogenic Diets had the potential for more nutritional inadequacies for both vitamins and minerals as compared to the other fad dietary patterns. The remaining seven dietary patterns had, on average, nutritional concerns about over the consumption of sodium and an inadequate consumption of vitamin E and vitamin D.

## 4. Discussion

The current study sought to determine the potential for improving dietary quality among popular fad dietary patterns. We first created a working definition of a fad diet based on both scholarly and non-scholarly sources; then, we determined the most popular fad dietary patterns in America based on the most searched “diet” term or phrase in Google. A secondary purpose was to evaluate the extent to which the selected popular fad dietary patterns adhered to the DGAs as measured, but with the HEI staying within the rules of the dietary pattern. Finally, we synthesized common elements from each of the included fad dietary patterns to determine how adherence to the DGAs could be maximized and, therefore, maximize the dietary quality of Americans. This evaluation and synthesis showed that some popular fad dietary patterns have the potential for a high dietary quality (HEI scores > 80) when the fad dietary patterns are planned carefully to follow the DGAs to the best extent possible under the rules of the diets. The fad diets that reached the cut-off point for high dietary quality included the Low-FODMAP Diet, Plant-Based/Vegan Diet, Military Diet, Fasting Diet, and the DGA-Compliant Diet. In comparison to the average HEI scores of the American population (59), the Ketogenic Diet (77), Plant-Based/Vegan Diet (84), Military Diet (82), Low-FODMAP Diet (89), Fasting Diet (86), and Paleo Diet (69) have the potential to achieve dietary quality scores that are higher than the current population average, highlighting the importance of understanding that there are many ways to achieve a high-quality diet. Other key findings suggest that nutritional adequacy is potentially problematic in the Carnivore and Ketogenic Diets, although the Ketogenic Diet can achieve a high HEI score. The remaining seven fad dietary patterns can meet most of the RDAs, when planned to follow the DGAs.

Table 6 suggests minor alterations that could be made to improve the dietary quality of popular fad dietary patterns that “meet people where they are” as a strategy to improve adherence to dietary patterns that are health-promoting. Note that these recommendations may not typically be allowed for each popular fad dietary pattern, if strict adherence to the parameter is the priority. For this synthesis, we grouped the popular fad dietary patterns in the following manner: least restrictive (DGA-Compliant Diet, Plant-Based/Vegan Diet, and Fasting Diet), moderately restrictive (Military Diet, Paleo Diet, and Low-FODMAP Diet), and most restrictive (Ketogenic Diet, Carnivore Diet, and Liquid Diet).

A limitation of the current study is that the sample meal plans were not isocaloric; however, the HEI analysis is standardized using foods/nutrients consumed per 1000 calories, and thus adjusts for the differences in calorie provision. However, for some popular fad dietary patterns, for example, the Liquid Diet, matching calorie content did not allow for a realistic application of this popular fad dietary pattern. Furthermore, the purpose of the current analysis was to determine how closely each popular fad dietary pattern could adhere to the DGAs to maximize HEI scores; therefore, rather than focusing on creating isocaloric menus for analysis, we determined that creating menus to maximize HEI scores was more aligned with the purpose of this study. Another limitation is that these example menus, designed to maximize dietary quality, potentially do not represent how individuals follow these popular dietary patterns. Rather, they are indicative of the potential for the fad dietary pattern to be considered high in dietary quality.

### Directions for Future Research

In alignment with the DGAC recommendations, we suggest that future research determines what dietary patterns Americans actually consume and to what extent they adhere to the rules/restrictions of the identified patterns. In doing so, recommendations might provide practical and reasonable insights, or the “low hanging fruit”, for improving dietary quality. Given that previous studies indicate a poor compliance to weight loss diets, future research should also aim to identify opportunities for altering restrictive diets to increase acceptability and adherence. Finally, future analyses should be conducted to determine what dietary patterns are emerging and gaining popularity, such that nutrition professionals can use best practices to help to determine how maximal dietary quality and nutrient intake may be achieved within preferred dietary patterns, or potentially with minor alterations that fall outside of the rules that would be acceptable to the client or patient.

## 5. Conclusions

This study suggests that there are many approaches to achieving better dietary quality. By using the preferred dietary pattern as a basis for a client/patient, making small changes within the rules or parameters of the dietary pattern might reveal acceptable strategies/components for improvement in dietary quality. Generally, popular fad dietary patterns might be viewed negatively, and there are many misconceptions regarding the “correct” way to eat. A dismissive stance from nutrition and public health professionals may overlook the potential of popular dietary patterns to be health-promoting.

## Figures and Tables

**Table 1:** Fad diet attributes.

Title, Author, Date	Source Type	Fad Diet Attributes					
		Lack of Physical Activity	Rigidity	Marketing	Restriction	Time	Other
Healthy weight, nutrition, and physical activity [8]	Website				X		unhealthy, typically fail
Staying Away from Fad Diets [9]	Website	X	X			X	
“Weight-loss diets”, 2018	Website		X		X	X	
Fad Diets vs. Healthy Behaviors [10]	Website		X	X		X	refers to food as “good” or “bad”
Nutrition for weight loss: What you need to know about fad diets [11]	Website	X	X	X		X	draws simple conclusions from complex medical research
A Consumer’s Guide to Fad Diets [12]	Textbook	X	X	X	X	X	claims individuals can “alter your genetic code” or “rest your metabolism” and fails to mention potential risks
The Latest and Greatest Weight-Loss Diet—Again [13]	Textbook			X	X	X	recommends liquids rather than foods and fails to inform the client of risks
Fad Diets [14]	Website	X	X			X	fails to mention health warnings
Fad Diets: Lifestyle Promises and Health Challenges [15]	Journal		X		X	X	may negatively impact health, propelled by an ideal body image and low self-esteem, may cause yo-yo dieting, and caused from ideal body type portrayed through media influence
Review of Dietary Practices of the 21st Century: Facts and Fallacies [16]	Journal		X	X		X	does not take into consideration social and environmental factors relating to dietary consumption, promotes weight loss rather than health, based on the cultural norms of the time
Functional Foods in fad diets: A review [17]	Journal			X		X	
Fad diets: Diet, types, tips [18]	Website		X	X	X	X	
Fad diets: The Slim on good nutrition [19]	Textbook	X		X		X	
Fad Diets and Obesity—Part IV: Low-Carbohydrates vs. Low-Fat Diets [20]	Journal				X	X	

Note. X denotes presence of fad diet attribute. Lack of physical activity refers to no component related to encouraging physical activity. Rigidity refers to a requirement of intake of certain foods. Marketing refers to the marketing of a product for sale or endorsements of the dietary pattern by celebrities. Restriction refers to a requirement of the complete omission of foods or food groups. Time refers to rapid timeframes for weight loss.

**Table 2:** Definition and parameters for the selected fad dietary patterns.

Dietary Pattern	Description
Ketogenic Diet	Dietary pattern characterized by a low-carbohydrate intake (<50 g/day), moderate protein intake (~20% of total caloric intake), and high fat intake (~70% of total caloric intake) [22].
Plant Based/Vegan Diet	The vegan diet is a plant-only diet that allows no consumption of animal products; the vegetarian diet is a plant-based diet where eggs and dairy may be consumed [23,24].
Fasting Diet	Umbrella term used to define dietary patterns where individuals refrain from eating for strategic periods of time followed by normal eating, and this process in repeated for potential health effects [25]. Alternate day fasting: 1 day o fasting followed by a day of eating. Alternate day-modified fasting: 2 days of fasting per week and 5 days of normal eating. Periodic fasting: numerous days of consecutive fasting per month. Time-restricted feeding: restricted eating window to certain hours of the day.
Carnivore Diet	No caloric restriction is necessary, and no fruits or vegetables are acceptable; the only permitted foods are meat, especially fatty cuts, and animal byproducts, like bone marrow or bone broth [26].
Liquid Diet	Diet consisting of the following permitted liquids: Water (plain, carbonated, or flavored); Fruit juices without pulp, such as apple or white grape juice; Bone broth; Miso; Strained vegetable juice; 100% fruit juice popsicles; Generally used for pre-operation situations where an empty colon is required and helps individuals to maintain hydration and electrolyte balance [27,28,29].
Military Diet	Three-day weight-loss program of rigid eating followed by four days of normal eating; options are available for individuals following a plant-based diet; no other beverages or foods are permitted [30].
Low-FODMAP Diet	Dietary pattern that uses the restriction of carbohydrate-rich foods, such as highly fermentable oligosaccharides, disaccharides, monosaccharides, and polysaccharides, to treat IBS, SIBO, and other gastrointestinal distress disorders; once symptoms subside, carbohydrate-rich foods are reintroduced into the diet as tolerated [31,32].
Paleolithic Diet	Dietary pattern that restricts the consumption of grains, legumes, peanuts, peas, dairy products, refined sugar, artificial sweetener, salt, potatoes, trans fats, vegetable oil, and highly processed foods and emphasizes the intake of fruits, vegetables, lean proteins, seafood, nuts, and seeds [33].

**Table 3:** Dietary pattern parameters.

Dietary Pattern	Rules	Timeframe for Recommendations	Calorie Intake	Vegetables (Cup Equivalent)					Fruits (Cup Equivalent)	Grains (oz Equivalent)		Dairy/Dairy Alternatives (Cup Equivalent)	Protein (oz Equivalent)			Oils (g)
				Dark Green	Red Orange	Legumes	Starchy	Other		Whole Grains	Refined		Seafood	Meats, Poultry, Eggs	Nuts, Seeds, Soy Products	
**HEI/DGA**		**Daily**	**2000**	**2.5**					**2**	3	3	**3**	**5.5**			**27**
		Weekly	14,000	1.5	5.5	1.5	5	4	14	21	21	21	8	26	5	189
**Keto Diet**	<50 g of carbs/day; 20% calories from protein; 70% calories from carbs	Daily	√	<50 g of carbohydrates per day									20% of caloric intake from protein sources			70% of caloric intake from fat sources
		Weekly	√													
**Plant-Based/Vegan**	No meat products	Daily	√	√					√	√	√	√	√ excluding animal sources for vegan; allowing eggs for plant-based			√
		Weekly	√	√	√	√	√	√	√	24.5	√	√	24.5 excluding animal sources excluding animal sources for vegan; allowing eggs for plant based			√
**Fasting Diet: Time-Restricted Feeding**	Feeding window—16 h fast: 8 h feeding	Daily	√	√					√	√	√	√	√	√	√	√
		Weekly	√	√	√	√	√	√	√	√	√	√	√	√	√	√
**Carnivore Diet**	Meat only, mainly red meats	Daily	√	0									All dietary intake from animal meats only, prioritizing red meats			
		Weekly	√													
**Liquid Diet**	Clear liquids only: fruit juices without pulp, bone broth, miso, strained vegetable juice, 100% fruit juice popsicles	Daily	√	√ Strained vegetable juice (25 cal and 1 serving per ½ cup)					√ Fruit juices or 100% fruit juice popsicles, all without pulp (60 cal and 1 serving per ½ cup)	0			Bone broth (80 cal and 5 g per cup) and miso (60 cal and 5 g per cup)			-
		Weekly	√	√	√	0	0	√	√							
**Liquid Diet**	Clear liquids only	Daily	√	√					√	0						
		Weekly	√	√	√	0	0	√	√							
**Military Diet**	Rigid dietary intake following plan (see meal plan below *) for 3 days followed by normal eating for 4 days	Daily (4 normal eating days)	√	√	√	√	√	√	√	√	√	√	√	√	√	√
**Low-FODMAP Diet**	Restriction of carbohydrate-rich foods, such as highly fermentable oligosaccharides, disaccharides, monosaccharides, and polysaccharides	Daily	√	Restriction of high-FODMAP vegetables: beans, lentils, broccoli, asparagus, garlic, and onion Inclusion of low-FODMAP vegetables: tomato, eggplant, and cucumbers					Restriction of high-FODMAP fruits: peaches, apples, and pears Inclusion of low-FODMAP fruits: blueberries, oranges, limes, lemons, strawberries, and pineapple	Restriction of high-FODMAP grains: wheat-based products Inclusion of low-FODMAP grains: oats, quinoa, rice, and gluten-free products		Restriction of high-FODMAP dairy: milk, yogurts, ice cream, and milk-based products Inclusion of low-FODMAP dairy: some cheeses and non-dairy-based milk products that are calcium-fortified	√	√	√	√
		Weekly	√										√	√	√	√
**Paleolithic Diet**	Restriction of grains, legumes, peanuts, peas, dairy products, refined sugar, artificial sweetener, salt, potatoes, trans fats, vegetable oil, and highly processed foods	Daily	√	√					√	0	0	0	√			√
		Weekly	√	√	√	0	0	√	√	0	0	0	√	√	√	√

Note. √ indicates where the recommendations are the same as those of the HEI/DGA. * Military Diet meal plan may be found in Section 3.7.

**Table 4:** Military diet meal plan.

Day 1	Day 2	Day 3
Breakfast: 1/2 grapefruit, 1 slice of toast, 2 tablespoons of peanut butter, 1 cup of coffee or tea (with caffeine) Lunch: 1/2 cup of tuna, 1 slice of toast, 1 cup of coffee or tea (with caffeine) Dinner: 3 ounces of any type of meat, 1 cup of green beans, 1/2 banana, 1 small apple, 1 cup of vanilla ice cream	Breakfast: 1 egg, 1 slice of toast, 1/2 banana Lunch: 1 cup of cottage cheese, 1 hard-boiled egg, 5 saltine crackers Dinner: 2 hot dogs (without bun), 1 cup of broccoli, 1/2 cup of carrots, 1/2 banana, 1/2 cup of vanilla ice cream	Breakfast: 5 saltine crackers, 1 slice of cheddar cheese, 1 small apple Lunch: 1 hard-boiled egg (or cooked however you like), 1 slice of toast Dinner:1 cup of tuna, 1/2 banana, 1 cup of vanilla ice cream

**Table 5:** HEI scores for the popular fad dietary patterns.

HEI Scores for the Popular Fad Dietary Patterns										
	Scoring Requirements for a Maximum Score per 1000 kcal	DGA Compliant Diet	Ketogenic Diet	Plant-Based/Vegan Diet	Fasting Diet	Carnivore Diet	Liquid Diet	Military Diet	Low-FODMAP Diet	Paleo Diet
**Adequacy Components (5 pt Scale)**										
Total Vegetable	≥1.1 cup equivalent	5	5	5	5	0	5	5	5	5
Greens and Beans	≥0.2 cup equivalent	5	5	5	5	0	2.1	5	5	5
Total Fruit	≥0.8 cup equivalent	5	3.5	5	5	0	5	5	5	5
Whole Fruit	≥0.4 cup equivalent	5	5	5	5	0	0	5	5	5
Total Protein	≥2.5-ounce equivalent	5	5	5	5	5	4.3	5	5	5
Seafood and Plant Protein	≥0.8-ounce equivalent	5	5	5	5	5	0	5	5	5
**Adequacy Components (10 pt Scale)**										
Whole Grains	≥1.5-ounce equivalent	6.4	2.9	7.4	5.6	0	0	7.1	8.2	0
Dairy/Dairy Alternatives	≥1.3 cup equivalent	10	3.7	10	10	0.04	0	8.7	7.8	0.4
Fatty Acids Ratio	(PUFAs + MUFAs)/SFAs Max ≥ 2.5 Min ≤ 1.2	7.18	10	10	10	10	5.6	2.6	10	10
**Moderation Components (10 pt Scale)**										
Sodium	Max: ≤1.1 g Min: ≥2.0 g	0	7.01	0.03	1.3	0	0	5.5	3.09	0
Refined Grains	Max: ≤1.8-ounce equivalent Min: ≥4.3-ounce equivalent	7.8	10	10	9.6	10	10	10	10	8.5
Saturated Fats	Max: ≤8% of energy Min; ≥16% of energy	10	4.8	10	10	0	10	8.37	10	8.5
Added Sugars	Max: ≤6.5% of energy Min; ≥26% of energy	9.8	10	10	9.33	10	10	9.8	9.99	10

**Table 6:** Strategy to improve dietary quality.

Areas of Concern Where HEI Scores Are Consistently Low	Modifications Made within the Study to Maximize HEI Scores	Popular Fad Dietary Patterns Grouped by Restriction Level (Low, Moderate, and High)	Modifications Suggested for Future Studies to Further Maximize HEI Scores	Example of Alterations	Common Elements to Further Maximize Scores
High intake of sodium and refined grains; low intake of whole grains.	Where applicable, incorporating different sources of lean proteins and plant-based protein sources, selecting a variety of different plant foods, reducing the intake of added sugar and sodium, incorporating different types of mono- and polyunsaturated fats, selecting low-fat dairy/dairy alternative products, and selecting whole grains over refined grains.	DGA-Compliant Diet, Plant-Based/Vegan Diet, and Fasting Diet	Selecting low-sodium foods and reducing the amount of refined grains while replacing them with whole grains.	Replacing high-sodium pretzels with either a reduced-sodium option or another non-refined grain.	Selecting products with a low-sodium content, incorporating more whole grains, and minimizing saturated fat intake.
High intake of sodium, refined grains, and saturated fats; low intake of whole grains and dairy/dairy alternatives.		Military Diet, Paleo Diet, and Low-FODMAP Diet	Selecting low-sodium foods, selecting products with limited saturated fats, and including more whole grains * and dairy/dairy alternatives *.	Replacing hot-dogs with a lean protein, substituting ice-cream with yogurt, and adding in whole grains where restricted.	
High intake of sodium, refined grains, and saturated fats; low intake of whole grains, fruits, vegetables, and dairy/dairy alternatives.		Ketogenic Diet, Carnivore Diet, and Liquid Diet	Adding whole grains * and dairy/dairy alternative products, reducing sodium intake, and balancing fat intake.	Incorporating milk or milk alternatives, selecting a low-sodium broth, substituting bacon for a lean protein, and adding whole grains where restricted.	

Note. * indicates where recommendations might fall outside of the rules of the popular fad dietary pattern.

## Data Availability

Data described in the manuscript, code book, and analytic code will be made available upon request pending application and approval.
